# Thermal Tomography Imaging in Photonic Traditional Chinese Medicine Information Therapy with Holistic Effect for Health Whole Nursing

**DOI:** 10.1155/2015/492391

**Published:** 2015-03-03

**Authors:** Binggang Ye, Zhouyi Guo, Hanchuan Huang, Xicheng Yang

**Affiliations:** Photonic Chinese Medicine Lab, College of Biophotonics, South China Normal University, Guangzhou 510631, China

## Abstract

A photonic traditional Chinese medicine (TCM) information therapy was developed that has applications in whole health nursing including the prevention and treatment of ischemic cardiovascular and cerebrovascular diseases as well as the conditioning of the subhealth state. This therapy utilizes the beam of a 630 nm LED light to irradiate the oropharynx, while simultaneously employing two beams of 650 nm LED light to irradiate corresponding acupuncture points resulting in a synergistic outcome. This method was named “1 + 2 phototherapy.” The principle mechanism of the therapy is a series of photon induced biological effects that are triggered by stimulating the photosensitive tissues of the oropharynx. This tissue includes the oral mucosa, capillaries, lymph nodes, saliva glands, nerves, and Jingluo and is stimulated by light beams of certain photon energy and imitative acupuncture information. Thermal tomography imaging shows that the average temperature of the upper-body was improved significantly after oropharyngeal irradiation under irradiation of “Futu point”: the heat radiation of the spine, as well as chest, shoulders, arms, and clavicle, increased under irradiation of “Hoku,” whereas the overall average temperature was below the temperature before irradiation. The experiment indicates that this therapy can promote blood circulation, regulate varied physiological parameters, and have holistic effects in whole health nursing.

## 1. Introduction

TCM has a profound dialectical therapy theory, which plays a unique role in rehabilitation. TCM believes that diseases can be divided into two categories: “deficiency syndrome” and “excessive syndrome.” Deficiency syndrome is caused by the basic material loss, for example, coronary heart disease, body weight loss, and fatigue, while excessive syndrome is caused by the invasion of pathogenic qi, for example, hypertension and high cholesterol. TCM also believes that the balance of yin and yang is a sign of the body in a healthy state. If this balance breaks, the body will be in a pathological state. Therefore, tonifying deficiency, treating excess with purgation, and balancing yin and yang are the essences of TCM theory. They are also the important principles to treat the diseases.

Ischemic cardiovascular and cerebrovascular diseases are common diseases which pose serious threats to human health [1, 2], especially in elderly populations. Subhealth (or subclinical) state is an intermediate stage between healthy living and a disease state [3, 4]. At present, the primary methods of treatment of ischemic diseases in clinical settings include drug therapy and interventional treatment [5]. It is well known that long-term medications can have unwanted side effects. Interventional treatment often has the problems of trauma, pain, and the possibility to be only carried out in hospital settings. Many treatment methods have the disadvantage of only relieving symptoms and are often expensive regimens that do not wholly cure the particular disease [6]. Often enough, these treatment regimens can be difficult to adhere to and may be rejected by patients. There are plenty of methods for conditioning one's subhealth state [7] such as drug therapy, exercise, meridian exercise, dieting, physical therapy, and other natural remedies. Unfortunately, though these therapies have more general applications, they lack a specific target and are restricted by the condition of the patient. Many patients cannot find a tailored treatment method for their disease state, leading to low efficacy of treatments. Therefore, there is an urgent need to develop a method for subhealth conditioning, prevention, and treatment of ischemic cardiovascular and cerebrovascular diseases that is more simple, effective, economic, and easy to adhere to for patients.

One kind of photonic TCM information therapy, developed by our institute, is simple, effective, economical, and easy to adhere to treat diseases. This therapy, founded on photonic TCM theory [8–11], is based on an organic combination of laser physical therapy, laser acupuncture, laser blood therapy, and integrated TCM information modulation techniques [12]. Furthermore, laser physical therapy mainly uses the low-energy laser stimulation on human skin and subcutaneous tissue, causing the body to regulate, promote, maintain, and restore the physiological functions, prevent disease, promote rehabilitation, eliminate inflammation, repair tissue, and relieve pain. These therapies are commonly used for all kinds of acute and chronic inflammation from sprains, strains, ulcers, and traumatic injuries [13]. Laser acupuncture, similar in method to acupuncture needles, can induce one's body to release many strong opioid peptides [14] and other biologically active substances into the central and peripheral regions of the body. These substances can play analgesic and anti-inflammatory roles while dilating blood vessels and many other effects [15] to treat migraine headaches, hypertension, periarthritis of the shoulder, and many other conditions. Laser blood therapy uses a low energy laser to irradiate human blood tissue. This therapy can improve blood parameters, promote microcirculation, improve immune function [16], and is often used for the treatment of cardiovascular and cerebrovascular disease, adjuvant treatment of respiratory diseases, endocrine diseases, autoimmune diseases, and diabetes [17]. The mechanisms of laser physical therapy, laser acupuncture, and laser blood therapy treatment are all derived from the principle of low-energy laser interaction with biological tissue [18]. These treatments differ slightly in their use. Laser physical therapy focuses on local trauma organization, laser acupuncture focuses on the human acupoints, and laser blood therapy focuses on blood tissue. The current use of low-energy laser irradiation therapy uses electromagnetic wave as physical treatment factor, but, in the actual applied process, it only makes use of the energy role of electromagnetic wave and ignores its role of carrying information. A number of experiments show that the pulsed laser is more conducive to stimulate the body's functional regulation mechanisms [18, 19], but this monotonous pulse signal cannot achieve symptomatic treatment, and long-time repeated stimulation will cause the body to adapt and reduce its efficacy. Reinforcing and reducing acupuncture therapy, summed up in long-term clinical practice, is able to produce special treatment efficacy precisely because it carries the different modulation information. This information can be confirmed to perform healthy regulation of physiological functions by a large number of clinical practices. With respect to the patient's physical condition, the use of reinforcing and reducing acupuncture information to modulate the laser beam, which adheres to the principles of TCM dialectical therapy, follows the TCM acupuncture principle of treating deficiency syndrome. Reinforcing and reducing acupuncture information also tonifies and treats excessive purgative syndrome by adopting the appropriate wavelength and appropriate strength low-energy laser beam to irradiate patient's oral and nasal blood rich zones and acupoints associated with the disease at the same time. Use of laser energy and information can trigger a variety of body tissues' adjustment mechanisms to restore the body's normal physiological functions. In traditional acupuncture theory, stimulating the specific acupoints can treat a variety of diseases. Such points are “Futu,” as an acupoint of the hand; Yangming, for the large intestine; and Jinluo, for treatment of stubborn hiccups, pseudobulbar palsy, back pain, hypertension, and other illnesses [20]. The acupoint “Hoku,” which is related to the qi transformation function of the whole body [21], has functions of reconciling blood, dredging meridians, dispelling wind, relieving extremities, resuscitation, relieving analgesic pain, and so on [22, 23]. According to the IEC Standard 825-1, an LED can be used as a low intensity laser and is proved to be safe to the human body [24]. In our study, this therapy adopts a beam of 630 nm LED light to irradiate the oropharynx, while utilizing two beams of 650 nm LED light to synergistically irradiate corresponding acupuncture point, referred to as “1 + 2 phototherapy” [25, 26]. With the selection of appropriate treatment methods and insisting on certain courses, one will attain a significant therapeutic effect. Such a method is expected to become a new means of conditioning subhealth, prevention, and treatment of the cardiovascular and cerebrovascular ischemic diseases.

## 2. Materials and Methods

### 2.1. Method of the Therapy

According to hemorheology theory, the fundamental reason of the generation of subhealth state and ischemic diseases is blood flow disorders. These diseases cause tissues and organs to have a lack of blood, lack of nutrients, oxygen, and be trapped in a state of ischemia and hypoxia [27]. In this therapy, the oropharynx is chosen as the main irradiation site. The LED light can easily irradiate through the mucous membranes and vessel wall and still effectively light the flowing blood within the vessels of the oropharyngeal mucosal capillary cloud. With the implementation of the light quantum activation and conditioning effect on the blood, it creates favourable conditions to promote blood circulation and tissue blood supply [28].

### 2.2. Photonic TCM Signal

**Figure 1:**
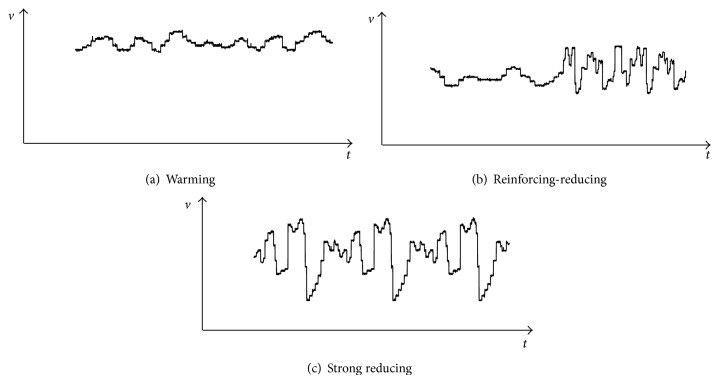
TCM signal waveform.

According to the traditional Chinese medicine reinforcing-reducing rule, the laser beam modulation is designed into multiple signal waveforms [26].  Figure 1 illustrates three typical signal waveforms. The warming signal can be applied to yin body and deficiency symptoms [29], the strong reducing signal suitable for yang body and fever symptoms [30], and the reinforcing-reducing signal suitable for neutral and physical symptoms or its combination therein [31].

### 2.3. Instrumentation

The Thermal Tomography Imaging device, produced by Beijing Bioyear Medical Device Company, was used to compare the body heat radiation status and to study the therapy on metabolism regulation. Through direct acquisition of heat radiation information, false color displaying different radiation intensities corresponding to the distribution of temperature, and computer analysis, TTM can reflect real-time changes in the metabolism of the cells and tissues of the body [32].

### 2.4. Sample and Procedure

The subject is a 23-year-old male student, and the irradiation power on oropharyngeal is about 15 mW and its wavelength is 630 nm. The irradiation power on the acupoint is about 25 mW; the wavelength of these lights is 650 nm. The experimental procedure consists of Tables 1, 2, and 3.

## 3. Results and Discussion

**Figure 2:**
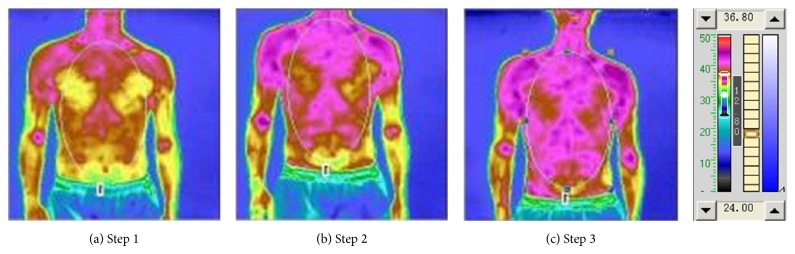
Front upper-body oropharyngeal irradiation TTM picture.

**Figure 3:**
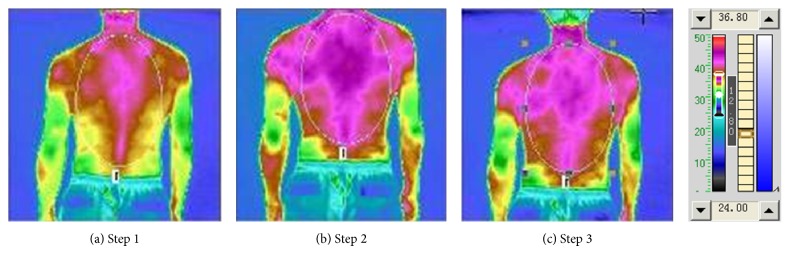
Upper-body oropharyngeal irradiation back TTM picture.

The photonic TCM information therapy implements and forms an overall adjustment treatment of “blood-based governance and comprehensive conditioning.” In the experiment Table 1, Figure 2 is the front upper-body oropharyngeal irradiation TTM picture, which illustrates the front upper-body thermal radiation status, Figure 2(a) is the result of TTM measurement before irradiation (Step 1), Figure 2(b) is the result of TTM measure 10 minutes after the oropharyngeal irradiation (Step 2), and Figure 2(c) is the result of Step 3.  Table 4 compares the temperature of the upper chest and abdomen under oropharyngeal irradiation. It can be clearly seen from Figure 2 that the thermal radiation level of the chest and abdomen was relatively low before irradiation. After 10 minutes of irradiation, the thermal radiation level was significantly increased and remained as such thereafter. After the second 10 minutes of irradiation, the thermal radiation level was also increased, but not as significant as the increase as the first 10 minutes of irradiation. From Table 4, one can see that the average temperature of the chest and abdomen rose up 1°C higher after 10 minutes of irradiation than that before irradiation. Also, the temperature increased slightly after the second 10 minutes of irradiation, which was not significant. Also the upper-body oropharyngeal irradiation back TTM measurements were recorded and the result of the comparison of the temperatures of the upper back chest and abdomen under oropharyngeal irradiation is as shown in Figure 3 and Table 5. As can be seen from Figure 3, after lighting for 10 minutes, the average temperature of the back also improved significantly, which increased by 0.55°C. There was a slight decline after the second 10 minutes of irradiation, and still 0.5°C higher than that before irradiation. According to TTM theory, the heat radiation image only reflects the heat radiation status of the surface of the body, but the heat radiation is not only generated by the cellular metabolism of the surface tissue, but also by the heat conducted from the tissue cells in vivo, so the heat radiation image can reflect the changing state of the body tissue cell metabolism. Generally, the enhancement of heat radiation indicates rushing metabolism, and the weakening of the thermal radiation suggests sluggish metabolism [33, 34]. This information can be used to assess the effect of metabolism regulation of the therapy by observing the changes in the thermal radiation image. As the surface thermal radiation is the inevitable product of the body's cell metabolism and blood circulation and the metabolism is dependent on blood circulation, the situation of surface thermal radiation distribution is a direct reflection of the status of the body's metabolism, as well as an indirect reflection of the status of the body's blood circulation.

**Figure 4:**
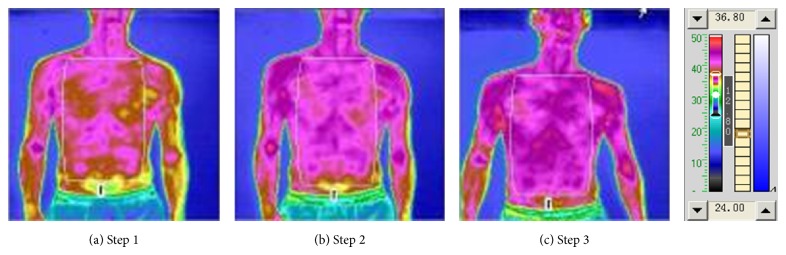
“Futu point” and oropharyngeal irradiation front TTM picture.

**Figure 5:**
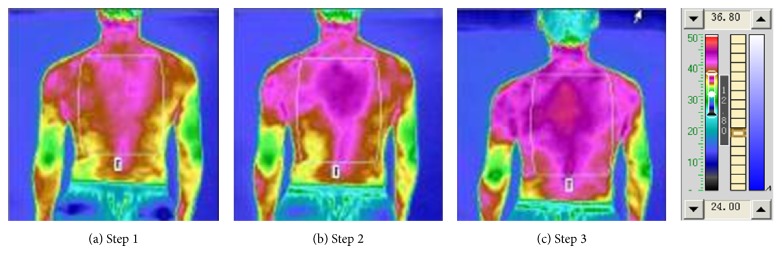
“Futu point” and oropharyngeal irradiation back TTM picture.

Figures 4 and 5 and Tables 6 and 7 are the results of the experiment Table 2 and Figure 4 with Table 6 illustrates the front upper-body thermal radiation status under irradiation of “Futu point” and oropharynx.  Figure 5 with Table 7 illustrates the back upper-body thermal radiation status. In Table 2, after the “Futu” irradiation, the area which changes more significantly is consistent with the hand Yangming large intestine meridian. The results indicate that the lighted acupuncture points changed on the presented meridian, which is similar to the results of traditional acupuncture. After irradiating the “Futu” and then lighting the oropharynx for 10 minutes, the average temperature of the front of the chest got a slight decrease, but the average temperature of the spine continued to increase, indicating the holistic adjustment effect of the acupuncture point irradiation. This suggests that the conventional laser treatments have local thermal effects, and it is unlikely to regulate overall blood circulation and metabolism. The photonic TCM information therapy can start nerve humoral regulation and meridians adjustment at the same time, so it has a function of overall regulation.

**Figure 6:**
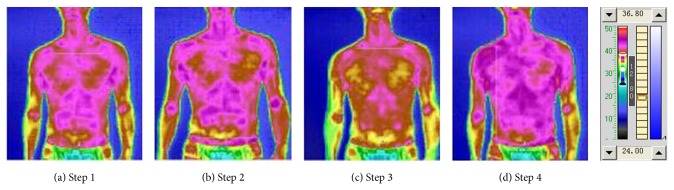
“Hoku point” and oropharyngeal irradiation chest and abdomen TTM picture.

**Figure 7:**
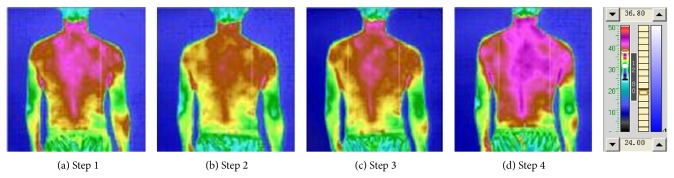
“Hoku point” and oropharyngeal irradiation shoulder and back TTM picture.

The results of the experiment Table 3 are given in Figures 6 and 7 and Tables 8 and 9. As can be seen from Figure 6 and Table 8, after irradiating “Hoku” for 10 minutes, the average temperature decreased by 0.3°C, but the maximum temperature dropped by 0.25°C; the minimum temperature rose by 0.05°C. It shows that, irradiating “Hoku” for 10 minutes, the overall temperature was lowered and reached some balance. The average temperature continued to drop after further irradiating “Hoku.” Then, after irradiating the oropharynx for 10 minutes, the overall recovery was enhanced and exceeded the temperature level of the irradiation before. In Figure 7 and Table 9, like chest and abdomen, the temperature of the back was also reduced after 10 minutes of irradiation of “Hoku.” After a further 10 minutes of irradiating “Hoku,” the average temperature was still below the temperature before irradiation in spite of a slightly rebound. The last 10 minutes of irradiation made the overall recovery enhanced and exceeded the level of the irradiation before. As a result, the thermal radiation of relevant region after 10–20 minutes of “Hoku” irradiation appears to be declining monotonically, or first declining then increasing. The declining is larger overall than the increasing effects, so the general trend is decreased overall, suggesting that the photon TCM therapy may have the function of bidirectional adjustment and balancing of yin and yang.

## 4. Conclusion

The results show that the photonic TCM information therapy plays a role in regulating blood circulation and metabolism, in regulating immune and endocrine systems, in diarrheal tonicity, and in balance of yin and yang. The present study suggests that the photonic therapy has a holistic effect in whole health nursing, which is used in the prevention and cure of ischemic cardiovascular and cerebrovascular diseases as well as in the conditioning subhealth state.

## Figures and Tables

**Table 1:** Observation of surface temperature change by “Futu” and oropharynx irradiation.

Experimental design	Responsibility	Remarks
*Step 1*. TTM measurement before irradiation *⇓* *Step 2*. After a 10-minute break, take TTM measurement after 10 minutes of the oropharyngeal lighting *⇓* *Step 3*. Repeat Step 2	Study surface temperature before irradiation Study surface temperature after two irradiation sessions	The continuous signal is adopted in the two irradiation sessions. Compare the three thermal radiation statuses

**Table 2:** Observation of surface temperature change by “Futu” and oropharynx irradiation.

Experimental design	Responsibility	Remarks
*Step 1*. TTM measurement before irradiation *⇓* *Step 2*. After a 10-minute break, take TTM measurement after 10 minutes of the “Futu” lighting *⇓* *Step 3*. After a 10-minute break, take a TTM measurement 10 minutes after the oropharyngeal irradiation	Study surface temperature before irradiation Study surface temperature after two “Futu” irradiation sessions	The pulse signal is adopted in the two irradiation sessions. Compare the three thermal radiation statuses

**Table 3:** Irradiating “Hoku” then oropharyngeal experiment.

Experimental design	Responsibility	Remarks
*Step 1*. TTM measurement before irradiation *⇓* *Step 2*. After a 10-minute break, take TTM measurement after 10-minute lighting of the right and the left “Hoku” *Step 3*. Repeat Step 2 *⇓* *Step 4*. After a 10-minute break, take TTM measurement 10 minutes after irradiation of oropharyngeal	Study surface temperature before irradiation Study surface temperature after two “Hoku” irradiation sessions Study surface temperature after irradiating oropharyngeal	The continuous signal is adopted in the three irradiation sessions, and the TTM images of chest, abdomen, and back in each experiment are taken. Compare the three thermal radiation statuses

**Table 4:** Comparison of the temperatures of the upper chest and abdomen under oropharyngeal irradiation.

Procedure	Av. temp. (°C)	High temp. (°C)	Low temp. (°C)
Step 1	30.60	31.70	29.00
Step 2	31.60	32.70	30.15
Step 3	31.85	32.70	30.15

**Table 5:** Comparison of the temperatures of the upper back chest and abdomen under oropharyngeal irradiation.

Procedure	Av. temp. (°C)	High temp. (°C)	Low temp. (°C)
Step 1	31.15	32.15	30.00
Step 2	31.70	32.70	30.65
Step 3	31.65	32.45	30.50

**Table 6:** Comparison of the temperatures of the upper front chest and abdomen under oropharyngeal irradiation.

Procedure	Av. temp. (°C)	High temp. (°C)	Low temp. (°C)
Step 1	31.45	32.45	30.00
Step 2	32.25	33.20	30.55
Step 3	32.10	32.95	30.90

**Table 7:** Comparison of the temperatures of the upper back chest and abdomen under oropharyngeal irradiation.

Procedure	Av. temp. (°C)	High temp. (°C)	Low temp. (°C)
Step 1	31.05	31.90	29.85
Step 2	31.90	33.10	30.15
Step 3	32.15	33.15	30.85

**Table 8:** Comparison of the temperatures of the chest and abdomen irradiation.

Procedure	Av. temp. (°C)	High temp. (°C)	Low temp. (°C)
Step 1	31.85	32.90	30.50
Step 2	31.55	32.65	30.55
Step 3	31.40	32.30	30.25
Step 4	32.20	33.05	30.70

**Table 9:** Comparison of the temperature of the shoulder and back irradiation.

Procedure	Av. temp. (°C)	High temp. (°C)	Low temp. (°C)
Step 1	31.40	32.35	29.80
Step 2	30.80	31.65	29.10
Step 3	31.10	32.00	29.25
Step 4	31.80	32.80	29.70
